# Linear and Non-Linear Soft Sensors for Predicting the Research Octane Number (RON) through Integrated Synchronization, Resolution Selection and Modelling

**DOI:** 10.3390/s22103734

**Published:** 2022-05-13

**Authors:** Tiago Dias, Rodolfo Oliveira, Pedro M. Saraiva, Marco S. Reis

**Affiliations:** 1 Department of Chemical Engineering, University of Coimbra, CIEPQPF, Rua Sílvio Lima, Pólo II—Pinhal de Marrocos, 3030-790 Coimbra, Portugal; tiagoagdias@gmail.com (T.D.); pas@eq.uc.pt (P.M.S.); 2 Petrogal, S.A., 4451-852 Leça da Palmeira, Portugal; rodolfo.oliveira@galp.com; 3 Dean of NOVA IMS, Campus de Campolide, Universidade Nova de Lisboa, 1070-312 Lisbon, Portugal

**Keywords:** Research Octane Number, catalytic reforming, dynamic soft sensors, data synchronization, data resolution

## Abstract

The Research Octane Number (RON) is a key quality parameter for gasoline, obtained offline through complex, time-consuming, and expensive standard methods. Measurements are usually only available a few times per week and after long delays, making process control very challenging. Therefore, alternative methods have been proposed to predict RON from readily available data. In this work, we report the development of inferential models for predicting RON from process data collected in a real catalytic reforming process. Data resolution and synchronization were explicitly considered during the modelling stage, where 20 predictive linear and non-linear machine learning models were assessed and compared using a robust Monte Carlo double cross-validation approach. The workflow also handles outliers, missing data, multirate and multiresolution observations, and processes dynamics, among other features. Low RMSE were obtained under testing conditions (close to 0.5), with the best methods belonging to the class of penalized regression methods and partial least squares. The developed models allow for improved management of the operational conditions necessary to achieve the target RON, including a more effective use of the heating utilities, which improves process efficiency while reducing costs and emissions.

## 1. Introduction

With the increased interest in Industry 4.0 solutions and in the opportunities emerging from big data scenarios, data-driven models, such as soft sensors, have been increasingly explored in industrial processes to take advantage of the huge volumes of industrial data that are being continuously collected and stored [1,2,3]. These models are instrumental for the optimized conduction of process operations, as well as for diagnostic activities focused on detecting problems and potential improvement opportunities. The quality of those models critically depends on the quality of data [4] and how the several challenges typical of industrial contexts are handled, such as the complexity of the systems under analysis, the high-dimensionality, presence of outliers, missing data, multirate and multiresolution measurements, time delays between different units, multiscale dynamics, etc.

Industrial processes are equipped with a wide diversity of sensors that allow the collection of a large number of variables alongside the process, primarily for the purposes of real-time monitoring and control [5,6,7,8,9]. These data often show patterns of correlation and effect sparsity that need to be handled properly during the development of inferential models or soft sensors. For instance, several variable selection and modelling algorithms (also known as wrapper methods) have been proposed with the goal of reducing data redundancy and selecting the more predictive regressors, such as heuristic stepwise selection regression methods (based on different criteria), genetic algorithms and best subset selection methods. On the other hand, latent variable methods accommodate redundancy through projection into low-dimensional subspaces. This is the case of principal component regression (PCR) [10] and partial least squares (PLS) [11,12,13]. PLS, in particular, is a methodology that has been extensively applied to develop inferential models in industrial scenarios due to its ability to handle collinearity, noise and a reasonable amount of missing data [12,14].The class of penalized regression methods can also deal with high-dimensional data. This class includes the Least Absolute Shrinkage and Selection Operator (LASSO) [15], Ridge Regression [16,17,18] and Elastic Net [19,20,21].

Another important aspect of industrial data is their sparse data structure due to the existence of missing data, different acquisition rates (multirate data), or operational/communication problems. The multirate scenario can occur when certain variables, for example, quality variables, are obtained less frequently due to the more complex measurement protocols, while other variables, such as process variables, are collected at very high rates through process sensors.

**Figure 1:**
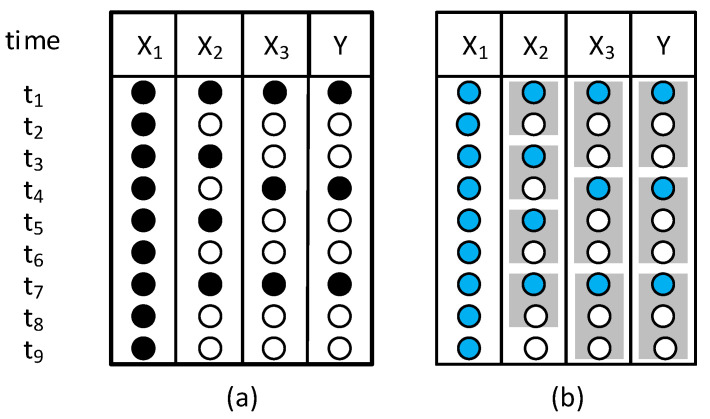
Schematic illustration of (**a**) multirate and (**b**) multiresolution data. A black circle represents an instantaneous measurement; a blue circle represents the aggregated value of several measurements. The grey rectangle represents the time window considered for each aggregation operation.

On the other hand, multiresolution (or multi-granularity) can also occur when the collected values have different levels of granularity in time. Their values, instead of representing instantaneous measurements, are the result of aggregation operations that merge observations with finer granularity into new ones with a coarser granularity. Quite often, these aggregations can occur by simple averaging operations. Figure 1 schematically depicts the differences between multirate and multiresolution scenarios: in the multirate scenario, the values represent the instantaneous measurement of the variables with different sampling rates (process variables $X_{1}$, $X_{2}$, and $X_{3}$ have a sampling rate of *t*, while Y has a sampling rate of 3*t*), whereas, in the multiresolution scenario, the values contain information with different levels of granularity (different resolutions). Regarding Figure 1b, in multiresolution data structures, the time window used for the aggregation is also called the time support. Thus, process variable $X_{2}$ has a time support of 2*t*, while Y has a time support of 3*t*. Although the two data tables may look similar, the values were obtained differently, and their meaning is also distinct. The concept of multiresolution or multi-granularity is often overlooked and still underexplored in data analysis [22]. However, it may have important implications for model development. Even when data are available at a single resolution, there is no assurance that the native resolution is the most adequate for model development. In fact, data collecting systems were designed and installed by third-party contractors, and their concerns were not to optimize the performance of future predictive models but to ensure that the relevant variables are sampled at a sufficiently high resolution and rate in order to control and monitor the system. Thus, it is important to include the selection of the modelling resolution as an aspect to be considered during the development of the inferential models [23].

In this work, we illustrate how the above-mentioned issues can be handled, in a systematic way, in the scope of a real process, catalytic reforming, which is one of the most important processes in petrochemical refineries. Catalytic reforming is the operation responsible for the conversion of low-octane naphtha into high-octane gasoline blending components, called reformates [24,25,26]. The Research Octane Number (RON) characterizes the resistance to knocking (or antiknocking tendency) of gasoline during its combustion in the engine and is a key quality parameter. If the gasoline RON is not within specifications, engines may not work properly, with significant consequences also on power loss and emissions. The goal of this work is the development of robust inferential models for predicting RON by considering different data granularities and assessing their impact on model accuracy. Furthermore, the alignment of the data from different units was also considered in each granularity scenario. The industrial process consists of a continuous catalyst regeneration (CCR) reforming unit from a major refinery located in Portugal. Using easy-to-measure process variables collected from the catalytic reforming process, our aim is to produce estimates of RON in real-time. With such an inferential model available, plant engineers can perform corrective actions earlier instead of waiting hours or even days for the laboratory results.

The rest of this article is organized as follows. Section 2 briefly describes the process analyzed in this work. Section 3 presents the main steps of the analytical workflow followed to develop soft sensors, including data collection, cleaning, pre-processing, and model development. The framework used to compare the different modelling alternatives is also described. Section 4 provides a brief summary of the different predictive methods that were tested and compared. Section 5 presents and discusses the main results obtained. Finally, Section 6 summarizes the main findings and conclusions of this work.

## 2. The Continuous Catalyst Regeneration (CCR) Unit

In this section, we present a brief description of the continuous catalyst regeneration (CCR) reforming unit. This unit converts linear paraffins present in the naphtha cut to higher reformate products for gasoline blending. This process essentially restructures the hydrocarbon molecules, transforming linear paraffins with low octane ratings into branched paraffins and aromatics [24,25,26].

**Figure 2:**
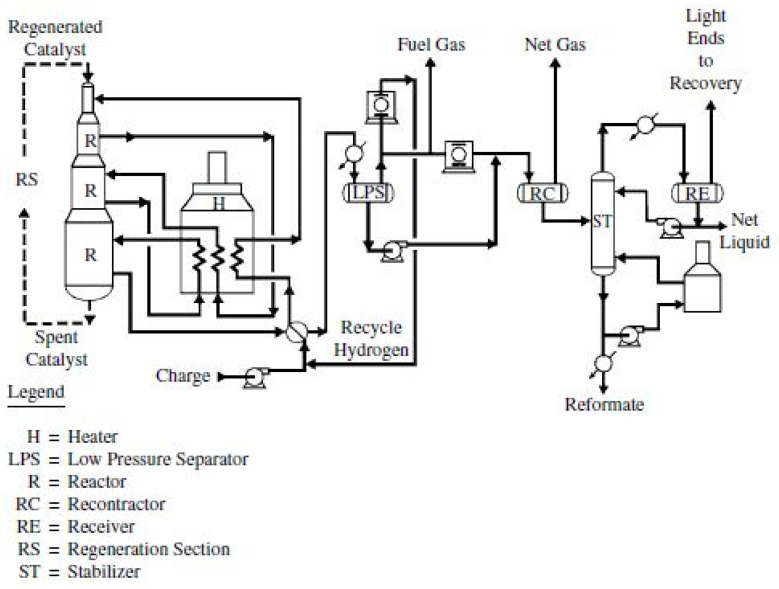
CCR Platforming process.

The CCR process is the most recent catalytic reforming technology. The main difference between CCR compared to other catalytic reforming units is the continuous addition of catalyst to the reactors, mitigating the effects of catalyst deactivation over time. A schematic representation of a CCR unit can be found in Figure 2. As in all catalytic reforming units, a preheating stage increases the temperature of the naphtha in the feed. The reactor is divided into three sections since different reactions occur at different stages. The final reaction products go to the separator section (LPS), where the heavier liquid (reformate) is sent to the stabilization column (“ST”) and recovered at the bottom. In addition to the reformate, another product of this unit is hydrogen, which is not only reused in this process but also directed to other processes in the refinery as well. The main goal is to deliver an exit stream with RON under specifications, despite the existing variation in the heavy gasoline stream entering the unit, and other causes of variability (e.g., due to environmental factors, operational actions, equipment-level events and degradation, catalyst modification, etc.), and at a minimum cost, both operational and for the environment (through reduced emissions), for instance avoiding the use of heat in excess in the furnace. For this reason, better control of RON is necessary, which requires more frequent access to good estimates of the RON level. Currently, RON measurements are made in the local laboratory through reference methods only a few times per week. Therefore, the development of inferential models appears as a valid alternative to improve the control of the unit. In the next section, we present the main steps of the workflow followed for developing inferential models for RON.

## 3. Data Analysis Workflow

As mentioned before, industrial data can raise a variety of analytical challenges. During the development of data-driven models, it is necessary to ensure that data have the required quality and the right structure to support the development of predictive models. Therefore, a framework suitable for this objective should be established that addresses all the relevant challenges that come with a particular industrial data set. In this section, we provide a brief overview of the main stages of such a workflow for inferential model development, as they were proposed in the literature.

Park and Han [27] described a three-step methodology for soft sensor design, including (i) preliminary process understanding, (ii) data pre-processing and analysis, and (iii) model determination and validation. The first stage is associated with finding a prior understanding of the process, its variables and existing interactions. In the second stage, the issues of outlier detection, noise reduction and data transformation are addressed. The final stage concerns the selection of the model structure, estimating its parameters and validating the model obtained with a new data set.

Alternatively, ref. [28] described a three-step strategy encompassing (i) data collection and conditioning, (ii) influential variable selection, and (iii) correlation building. The first step is related to the collection of data, an understanding of the problem, and assessing the relevance of the variables gathered. In addition, an outlier detection strategy needs to be implemented to exclude observations that do not represent valuable information about the process. It is also important to ensure that data are representative of the process for which the model is built. In the second step, a variable selection strategy is conducted to reduce the number of variables for analysis. The final step concerns the development of the predictive model.

In [5], several practical aspects of soft sensors are presented and organized in a five-step procedure, including (i) the selection of historical data from the plant database, (ii) outlier detection and data filtering, (iii) model structure and regression selection, (iv) model estimation, (v) and model validation. The first step involves a rigorous selection of the inputs that are to be collected. In the second step, an overview of the entire data set is performed for detecting and removing outliers and dealing with the presence of missing data. The following steps are related to the development of the model, starting with the selection of the model structure (linear or non-linear). Finally, the model is trained and validated using a new set of data.

Another five-step methodology was proposed by [29], involving (i) data inspection, (ii) data selection and steady-state identification, (iii) data pre-processing, (iv) model selection, training, and validation, and (v) soft sensor maintenance. This methodology starts with a first inspection of the structure of the data and the variability of the response variables. The second step is devoted to the selection of data (variables) and time frame (period of time for analysis). The data pre-processing step has the objective of dealing with missing data, the detection of outliers, feature selection, and accommodation for the existence of different sampling rates. The next step is focused on the selection of the model structure, which can also benefit from past experience. Once the model is estimated, it is always necessary to assess its performance on an independent data set. After developing the inferential model, it is important to perform its maintenance and to retune its parameters on a regular basis to overcome potential process and instrumentation drifts.

**Figure 3:**
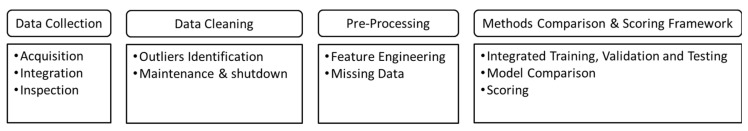
Data analysis workflow for inferential model development followed in this work.

The different proposals for data analysis workflows presented above share some communalities. More specifically, it is possible to identify four stages that they have in common, which include gaining process insight and data collection, data cleaning, data pre-processing, and model estimation and validation. These stages are presented in Figure 3 and will be followed in this work, with some adaptation. In the following subsections, we describe the operations conducted in each stage of the refinery case study.

### 3.1. Data Collection

In this stage, the different data sources are accessed, brought to a centralized repository and integrated. All of the data were initially transferred to Microsoft^®^ Excel using a Visual Basic for Applications (VBA) code. The subsequent analysis was carried out in the MATLAB^®^ environment (The MathWorks, Inc., Natick, MA, USA).

The CCR data set was obtained from the CCR unit of the GALP fuels plant at the Matosinhos refinery, located in Portugal. This data set contains 1,048,320 records, spanning an extended period of 24 months and conveying information from different process streams and zones (feed, reaction, separation, etc.), such as flows, temperatures, and pressures. Information regarding the CCR data set originated from two sources: process variables and product quality variables. The process variables are linked to the process operation and contain sensor measurements, such as flow rates, temperatures, and pressures. The product quality variable of interest is RON, which was obtained in the laboratory following a standard procedure, the ASTM Method D2699. Process variables consist of easy-to-measure variables collected every minute (temperatures, flows, pressures), whereas RON is measured once per day (or even less). For that reason, the data set has a sparse, multirate structure and may include outliers as well as missing data. Process engineers have informed that there were no periods of non-operation (e.g., maintenance or shutdown periods) over the 24 months under analysis.

### 3.2. Data Cleaning

The data cleaning stage is focused on detecting and removing bad data segments, as well as single out outliers to be more carefully scrutinized during a second pass, eventually with the support of process experts. Outliers are values that strongly deviate from the normal range of each variable or from their local correlation patterns [29,30]. Identifying and removing outliers is of extreme importance in industrial data analysis since they can introduce biases in descriptive statistics and inferential methods, as well as deteriorate the performance of predictive models. However, one should keep in mind that sometimes such unusual occurrences can provide useful insights into unexplored operational domains.

In this work, two categories of outliers were considered: global outliers and contextual outliers. Global outliers are values that fall outside of the feasible range of the sensor measurements. They are identified by applying simple variable-dependent thresholds called operation limits. Each variable has its respective operating range, and any value standing beyond that interval should be taken as an error and removed. This thresholding procedure was executed by using the information provided by the plant process engineers.

Contextual outliers are more difficult to identify since they comply with the technical range of the sensors but significantly deviate from the local pattern of dispersion. There are several techniques proposed to identify this type of outlier, such as the 3σ rule [31], the Hampel identifier [5,8,32], and the modified Hampel identifier with a moving window technique [32]. We started by applying the 3σ rule and the Hampel identifier but concluded that neither of them was able to detect most of the existing outliers since the thresholds selected by these methods are contaminated by the outliers they are aimed to remove. On the other hand, the modified Hampel identifier with a moving window technique was found to be a more robust option. It reduces the influence of the outliers since it does not consider the data set as a whole but only the local variability. The selection of the window size was determined variable by variable, alongside expert input from plant engineers, who also confirmed that the points removed were indeed outliers and not representative of real process operations.

In addition to outliers, excessive noise can also present a problem. Noise can be filtered out in order to improve the quality of the information collected [33]. Alternatively, the noise characteristics can be taken into account in the models by using uncertainty-based approaches [14,34,35].

### 3.3. Pre-Processing

In the pre-processing stage, some structural aspects of the cleaned data set are fixed to prepare it for further data analysis. The main aspect that needs to be resolved is the multirate nature of the data set. Another relevant topic of interest is related to missing measurements caused by transmission problems and other sensor/process malfunctions, which also contribute to the sparsity of the data set. Both aspects are covered in the pre-processing stage, which includes two sub-tasks: (i) the selection of the time resolution (granularity) for conducting data analysis and (ii) missing data imputation.

#### 3.3.1. Selecting the Resolution for Data Analysis

As stated above, the data set is composed of process variables (collected every minute) and the target response variable (collected, at best, once per day). This mismatch in acquisition rates limits the amount of dynamic information that is possible to infer from the data (according to the Nyquist theorem) and generates a sparse data structure that raises many problems for model building, as the observations where regressors are available do not match those where the response is known. These two sets of variables carry information with different levels of detail about the process, and therefore it is necessary to first establish a common resolution level for both process and quality variables.

The time resolution, or granularity, of a given data representation, is defined as the length of the non-overlapping time windows over which measurements are aggregated and summarized in some suitable way (e.g., using the mean, median, etc.). The resolution level should be selected by taking into account the goal of the data analysis and the structure of the data; see [4]. In this work, the objective was to develop an accurate predictive model for the estimation of RON. Therefore, it was first necessary to select a common resolution level that matches the available information (namely, the lower acquisition rate of RON). This procedure will not only bring all variables to a common resolution level but also reduce the size of the data to be analyzed and align the different data sources while minimizing the multirate sparsity present in the raw data. The mathematical operator used for the aggregation was the median of the values in the aggregation windows. The median was used instead of the mean, given its superior properties of robustness under the presence of atypical observations, which is a useful feature when quickly analyzing large amounts of industrial data.

**Figure 4:**
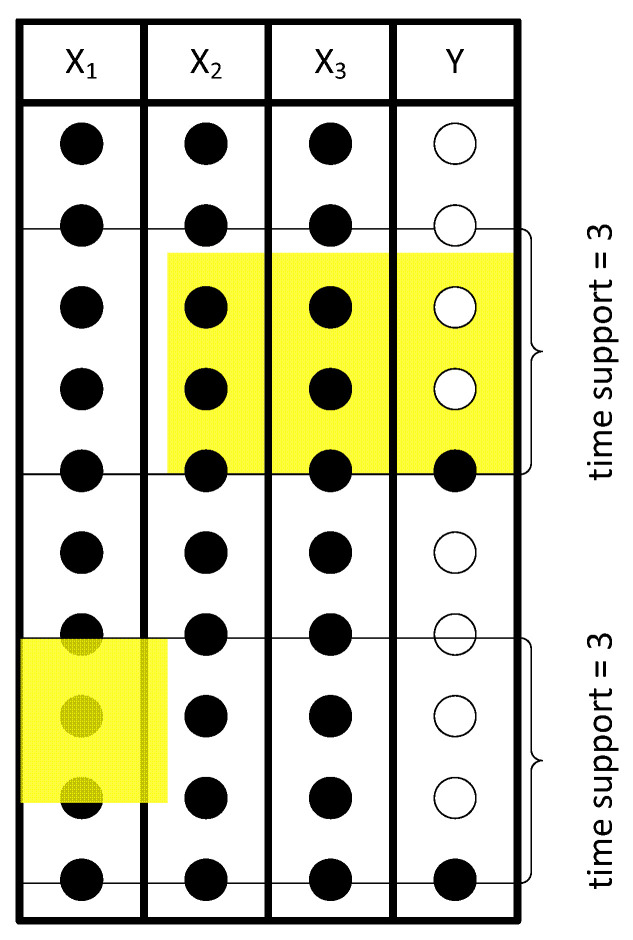
Representation of the S-SR[*t_s_*] methodology with a time support of three hours (S-SR3). It is assumed that each black circle contains a data value, whereas a white circle does not. The sampling time in this schematic illustration is 1 h.

Another important aspect of industrial data analysis is synchronization. As variables are collected from different units, the time delays between them need to be addressed. In this work, we have developed a methodology where the influence of data resolution and synchronization on the prediction performance of the inferential models are analyzed together. Five different synchronized single resolution scenarios were considered, each one of them with a different time support, $t_{s}$, referred to as S-SR[*t_s_*]). The aggregation was based on the time of occurrence of the output variable and then considering a time window of size $t_{s}$ towards the past. Therefore, if a sample for a RON measurement was collected at time $t_{\mathrm{RON}}$, the aggregation of the data is performed between $\left(t_{\mathrm{RON}}-t_{s}\right)$ and $t_{\mathrm{RON}}$ (i.e., it is synchronized with the response). The aggregation operation is the median of the value for each variable during the time period in question. The time supports (or window sizes) considered were the following: 24 h (S-SR24), 4 h (S-SR4), 3 h (S-SR3), 2 h (S-SR2), and 1 h (S-SR1). Figure 4 schematically depicts scenario S-SR3; since the time support is equal to three, with all the aggregation windows between $\left(t_{\mathrm{RON}}-3\right)$ and $t_{\mathrm{RON}}$. Taking the example illustrated in Figure 4 into consideration, the pre-processed data set resulting from the S-SR3 methodology has only two observations (only two values for Y are available), and they are then synchronized with the response. The first aggregation period takes place between $t_{2}$ and $t_{5}$, and the second one between $t_{7}$ and $t_{10}$. Independently of the size of the time support, the resulting data set always had the same number of observations because it was constructed based on the number of RON’s observations.

#### 3.3.2. Missing Data Imputation

There are several reasons for the existence of missing values in a data set. The most common are related to (i) maintenance and shutdown periods, (ii) sporadic failures and physical malfunctions of the sensors (since the sensors are physical devices, they may experience periodic failure conditions), and (iii) errors related to transmission issues between the sensors and the data server. While the periods of type (i) can be removed from the analysis, the periods of type (ii) and (iii) need to be handled through suitable imputation schemes. These are random sources of missing data patterns that may occur during process operation (note the difference with the deterministic nature of blanks caused by multirate collection systems).

Missing data imputation strategies estimate sequences of missing data by exploiting the existence of associations: either between variables (cross-correlation) or over time (autocorrelation). Various methods can be found in the literature that take advantage of the existence of cross-correlations under both missing at random (MAR) and missing completely at random (MCAR) scenarios, which are frequently based on expectation-maximization (EM) approaches [36,37,38,39]. In the present case study, the CCR unit is composed of several large pieces of equipment that constitute massive inertial elements characterized by large dynamic time constants. At the same time, the data are collected at very fast acquisition rates (every minute). In combination, these two conditions generate strong autocorrelation patterns. This is, therefore, the dominating association pattern, and it was the one explored in this work to estimate the scattered missing data. Autocorrelation represents the degree of correlation between a given time series and a lagged (i.e., delayed in time) version of itself. Given measurements, $Y_{1},Y_{2},\ldots ,Y_{N}$ at times $t_{1},t_{2},\ldots ,t_{N}$, the autocorrelation function, for lag k, is defined by Equation (1).

(1) $$r_{k}=\frac{\sum_{i=1}^{N-k}\left(Y_{i}-\bar{Y}\right)\left(Y_{i+k}-\bar{Y}\right)}{\sum_{i=1}^{N}{\left(Y_{i}-\bar{Y}\right)}^{2}}$$

**Figure 5:**
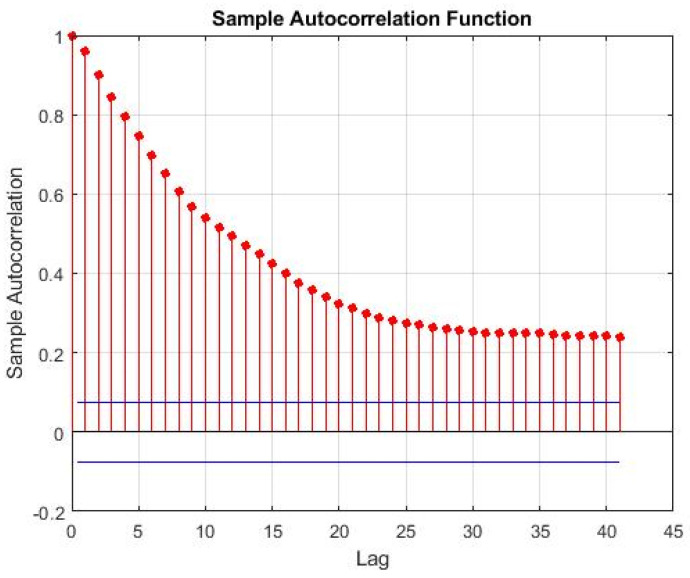
Autocorrelation function for variable X_1_.

For the sake of illustration, Figure 5 presents the autocorrelation for a given (real) process variable of the CCR unit, say X_1_, over the first 41 time lags, pointing out the strong autocorrelation pattern of this process variable (that is also present in others).

Since the dominant association structure present in our dataset corresponds to the variables’ autocorrelation, we have used interpolative schemes to estimate the missing data. The imputation was performed by a moving window technique, where missing data at the center were replaced by the median of the data points falling within the moving window [30]. This process was repeated for each variable. As happened in the stage of outlier detection, the validity of the final result from the imputation operations was confirmed by process engineers. EM methods could also be used to exploit correlation and autocorrelation. However, the interpolative method is simpler, computationally more scalable, and showed satisfactory accuracy.

### 3.4. Model Comparison Framework

During the design of an inferential model, it is important to select the most appropriate modelling strategy. However, it is not possible to make such a selection a priori; neither is it recommended to simply adopt the methods the user is most familiar with or that were successfully applied in other unrelated problems. The strategy followed in this work consists of comprehensively analyze the prediction ability of a carefully selected variety of methodologies and systematically compare their performances. This section describes how the pool of methods were compared. References are also made to additional insights that can be extracted from the models estimated.

In the proposed analytical pipeline, the set of predictive models (see Section 4) are compared through a protocol that combines: (i) Monte Carlo Double Cross-Validation [40,41,42,43,44,45] for robust estimation of the methods’ hyperparameter(s) and robust prediction assessment, (ii) statistical hypothesis to rigorously assess the methods’ relative performances, and finally (iii) a scoring operation, to summarize the results of the pairwise comparison tests in an easily interpretable ranking of performances. It is important to mention that no method from the classes referred above is expected to always perform better than the others and claim overall predictive superiority. Therefore, the final decision about which method to use should be based on a rigorous consideration of all the options available, conducted case-by-case, which further justifies the adoption of a comparison approach such as the one followed in this work. Even when the choice is not obvious, the decision process can only benefit from the outcomes of such a comparative analysis. Thus, a state-of-the-art comparison methodology based on Monte Carlo Double Cross-Validation was implemented in order to establish rankings of the best methods to adopt for addressing a particular problem, such as the one described in this work.

The methodology, described in Table 1, starts by defining the number of Monte Carlo runs to be conducted ($n_{MC}$) in the outer cycle of the framework, i.e., the number of times the internal operations will be repeated (in this work, we adopted $n_{MC}=25$). The internal operations consist of randomly splitting the data set into a training and testing set (step 1.a). The training set is then used to select hyperparameter(s) using 10-fold cross-validation (step 1.b), and a model is built using the training set (step 1.c) to predict the test set, after which the prediction errors are saved (step 1.d). The hyperparameter(s) for each method and more details on how they were optimized in step 1.c can be found in in Appendix B, Table A1.

Concerning the pseudo-code presented in Table 1, some decisions need to be made regarding the splitting (step 1.a) and the tuning of the hyperparameter(s) (step 1.b). For the splitting of the data, described in step 1.a, an 80/20 ratio was established. The splitting of data can occur in three ways: (i) order split, (ii) random split, (iii) and random stratified sampling split. In the case of order split, the first 80% of the samples go to the training set, while the remaining ones go to the testing set. The problem with this strategy is that it does not always provide a balanced representation of all of the conditions where the model should be trained. Therefore, in this work, we have adopted a random stratified sampling split approach, which consists of splitting the response variable into a pre-selected number of intervals based on its percentiles (e.g., 0–25, 25–50, 50–75, and 75–100 percentiles). Then, from each group, 80% of the data were randomly selected to form the training set, and the rest went to the testing set.

The training set in each run is used to optimize the selection of the hyperparameter(s) of each model (see Appendix B for the ranges considered for tuning the hyperparameters of the different methods). Since, in some industrial processes, it may be difficult to obtain sufficient historical data to develop a model, it is advantageous to use K-fold cross-validation (K-F CV) for this task. By default, 10-fold cross-validation was used (step 1.b). From the existing ten folds, nine are retained to train a model, and the remaining fold is used to perform the cross-validation. This process is repeated ten times, ensuring that each fold is used once as a validation set. This 10-fold cross-validation represents the inner cycle of (step 1.b). The RMSE on the left-out folds, obtained for each possible value of the hyperparameter(s), is saved, and the one leading to the lowest RMSE value is adopted for establishing the model hyperparameter(s).

Finally, the prediction errors on the left out test set are calculated and the root mean squared error $\left({\mathrm{RMSE}}^{\mathrm{test}}\right)$, given by Equation (2), is saved, as well as the coefficient of determination for the test set $\left(R_{\mathrm{test}}^{2}\right)$, given by Equation (3).

(2) $$\mathrm{RMSE}=\sqrt{\frac{\sum_{i=1}^{n}{\left(y_{i}-{\hat{y}}_{i}\right)}^{2}}{n}}$$

(3) $$R^{2}=\frac{\sum_{i=1}^{n}{\left({\hat{y}}_{i}-\bar{y}\right)}^{2}}{\sum_{i=1}^{n}{\left(y_{i}-\bar{y}\right)}^{2}}=\frac{\sum_{i=1}^{n}{\left(y_{i}-\bar{y}\right)}^{2}-\sum_{i=1}^{n}{\left(y_{i}-{\hat{y}}_{i}\right)}^{2}}{\sum_{i=1}^{n}{\left(y_{i}-\bar{y}\right)}^{2}}=1-\frac{\sum_{i=1}^{n}{\left(y_{i}-{\hat{y}}_{i}\right)}^{2}}{\sum_{i=1}^{n}{\left(y_{i}-\bar{y}\right)}^{2}}$$

where, $y_{i}$ is the ith observed response, ${\hat{y}}_{i}$ is the corresponding estimate, and n stands for the number of observations in the testing set. $\bar{y}$ is the mean of y, $\sum_{i=1}^{n}{\left(y_{i}-\bar{y}\right)}^{2}$ is the observed variability, $\sum_{i=1}^{n}{\left({\hat{y}}_{i}-\bar{y}\right)}^{2}$ is the variability explained by the estimated model and $\sum_{i=1}^{n}{\left(y_{i}-{\hat{y}}_{i}\right)}^{2}$ is the variability not explained by the model (residual variation). Since the outer cycle can be performed multiple times, it is possible to characterize the individual performance of the methods through their distributions of ${\mathrm{RMSE}}^{\mathrm{test}}$ (lower values suggest improved predictive performances).

An important aspect to ensure in this comparison framework is the following: in each run, the training and testing data sets are exactly the same for all of the methods under comparison. Therefore, the results are naturally organized in a pairwise fashion, making it possible to compare the different methods using statistical hypothesis tests with improved statistical power (as certain variation sources are blocked, in this case, the resampling variation). This comparison is performed through paired *t*-tests (given the high number of runs, the Central Limit Theorem assures the convergence of the mean to a Gaussian distribution, justifying the adoption of this test). The null hypothesis states that the mean difference between the two methods under comparison is zero (i.e., the means of ${\mathrm{RMSE}}^{\mathrm{test}}$ for the two methods are equal). The null hypothesis is rejected whenever the *p*-value obtained is lower than the adopted significance level (in this case, the significance level was set to α=0.05). To facilitate the analysis of the relative performance of the methods resulting from the battery of pairwise statistical tests, a scoring system was implemented. For each pair of methods under comparison, a score of 1 (“wins”) is given to the method with statistically significant lower $\bar{{\mathrm{RMSE}}^{\mathrm{test}}}$ (e.g., better prediction performance). A score of 0 (“loss”) is given to the method with statistically significant higher $\bar{{\mathrm{RMSE}}^{\mathrm{test}}}$ (e.g., worse prediction performance). In case the prediction performance of the two methods is not statistically distinct, a “draw” has occurred.

If a “draw” occurs, it is not clear which score should be attributed, and any value in the interval ]0,1[ could be arbitrarily chosen. By specifying a value in this interval, the performance of each method is obtained from the sum of the scores obtained in all pairwise comparisons, which would be a reasonable Key Performance Indicator (KPI).

However, as this sum depends on the actual score attributed to the “draws”, and any specific value would be debatable, we have computed the average KPI for all possible weights on the interval ]0,1[. More specifically, we have calculated two KPIs for the relative performance of each method: the mean KPI and the mean RANK, defined as follows:

**Mean KPI**—the average of the sum of scores when the “draw” scores span the interval ]0,1[

(4) $${\bar{\mathrm{KP}I}}_{m}=\frac{1}{1-0}\int_{0}^{1}KPI_{m}\left(s\right)ds=\int_{0}^{1}KPI_{m}\left(s\right)ds$$

**Mean RANK**—the average rank (in the descending ordering of performance) obtained when the “draw” scores span the interval ]0,1[

(5) $${\bar{\mathrm{RANK}}}_{m}=\frac{1}{1-0}\int_{0}^{1}RANK_{m}\left(s\right)ds=\int_{0}^{1}RANK_{m}\left(s\right)ds$$

In the implementation of this methodology, autoscaling (or z-score transformation, using the mean and standard deviation of the training set) was applied to all variables in each run of the outer cycle, to avoid any bias in the estimation of the testing set.

## 4. Predictive Modelling Methodologies

There are many regression methodologies currently available to perform predictive modelling. They can be just variants of the same base approaches but can also present great differences in their assumptions (e.g., regarding collinearity, sparsity, non-linearity, etc.) or estimation procedures which, consequently, lead to differences in the final outcomes. In this work, we adopted a selection of 20 regression methods that represent the main classes currently adopted for industrial data analysis; see also [42], guaranteeing in this way that the analytical landscape is covered in a balanced fashion. The following linear and non-linear regression methods were considered (a brief overview of each method is presented in Appendix A):

**Multiple Linear Regression (MLR):** [16,46] with and without variable selection (forward stepwise regression, FSR) [47,48,49].

**Penalized Regression Methods:** ridge regression (RR) [16,17], least absolute shrinkage and selection operator (LASSO) [15] and the elastic net (EN) [19,20,21].

**Latent Variable Methods:** principal component regression (PCR) [50,51,52,53], principal component regression with a forward stepwise selection strategy (PCR-FS) and partial least squares (PLS) [54,55,56,57,58,59,60,61,62].

**Tree-Based Ensemble Methods:** bagging of regression trees, random forests and boosting of regression trees [63,64,65,66,67].

**Artificial Neural Networks:** several backpropagation algorithms were considered [68,69]: Levenberg–Marquardt backpropagation (LM) and resilient backpropagation (RP); see also [68,70,71,72,73]; for applications in chemical engineering see [74,75,76,77].

**Kernel Latent Variable Methods:** Kernel PLS (KPLS) [78,79] and Kernel PCR [64,80,81], using the following kernel functions: Gaussian radial basis function and the polynomial kernel [82].

**Support Vector Machines Regression (SVR):** several kernels were tested: linear, polynomial, and Gaussian kernels [83,84,85,86,87].

## 5. Results

In this section, we report a summary of the results obtained, with a special focus on the comparison of performances for the different predictive methodologies used to estimate the industrial RON values for a major petrochemical facility.

As mentioned in Section 3, the CCR data set is composed of 1,048,320 samples from 41 process variables, such as the temperatures, flows, and pressures that originated in different locations (feed, reaction, separation, and utility zones) and streams of the CCR unit. These samples cover an extended period of 24 months. Table 2 provides the number of samples collected of RON values for the CCR data set, as well as the corresponding range of values.

### 5.1. Data Acquisition and Inspection

**Figure 6:**
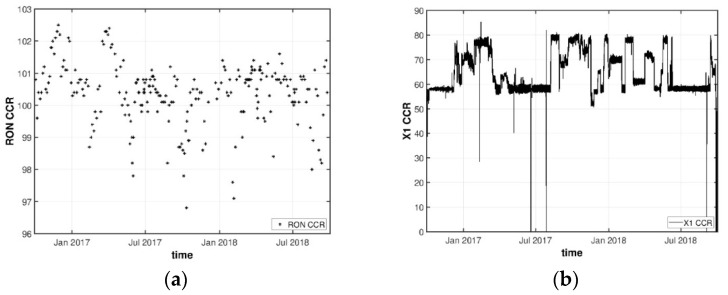
(**a**) Time series plot of RON during the data collection period; (**b**) Time series plot of $X_{1}$ during the data collection period.

There is a well-defined multirate structure in the CCR data set, with two different types of variables having distinct sampling rates: process variables were collected every minute while the target quality variable (RON measurements) are available, at best, once per day. Each recorded value regards an “instantaneous” observation (high resolution). Figure 6 depicts the time series plot of RON and process variable $X_{1}$ (process variables are anonymized to protect critical industrial information).

Analyzing Figure 6a, it is possible to verify that there are no observations below the limit value of the product quality (RON = 95). From Figure 6b, we can confirm that there are no non-operation periods due to plant shutdown or maintenance (this conclusion was validated by process engineers). From Figure 6b, it is also possible to verify the existence of outliers in the data set for this variable. The following section provides the results for the data cleaning step, where outliers are detected and handled.

### 5.2. Data Cleaning

**Figure 7:**
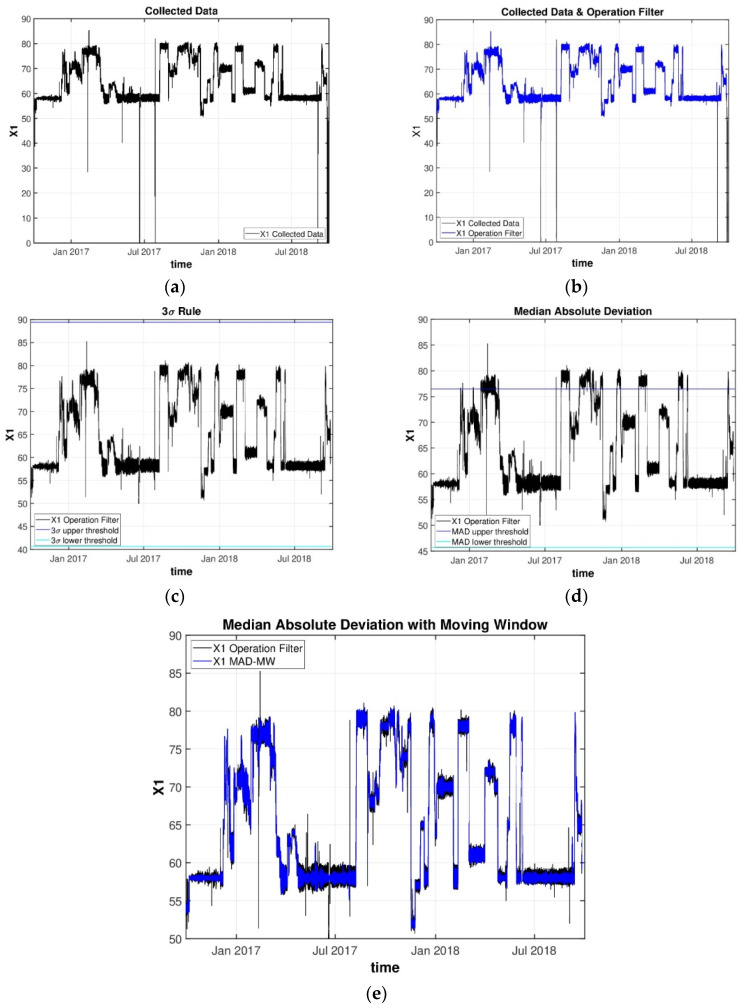
Comparison of various cleaning steps over the same variable X_1_: (**a**) No cleaning filter; (**b**) Operation filter; (**c**) 3σ filter; (**d**) Hampel Identifier; (**e**) adaptive Hampel identifier with moving window. The black lines for (**c**–**e**) are the same as the blue line from (**b**), i.e., the output of the operational filter.

As described before, several data cleaning filters were conducted over the data set with the objective of identifying and removing bad data segments and non-operation periods (e.g., shutdown or maintenance). For illustration purposes, Figure 7 presents the results obtained in the cleaning stage of the data analysis workflow for variable $X_{1}$ (the black line in this plot represents the collected data).

Global outliers were detected by applying an operation filter for each process variable. Each process variable has its own operating limits, and Figure 7b presents the data for variable $X_{1}$, after this stage was completed (blue line). From Figure 7b, it is possible to confirm that there are still several outlying observations that were detected. These points correspond to contextual outliers. To remove the contextual outliers, three strategies were studied, namely the 3σ rule, the Hampel identifier, and the modified Hampel identifier with a moving window technique. From Figure 7c,d, it is possible to verify that neither of the former two approaches was able to detect a large fraction of the existing outliers since their thresholds are influenced by the existence of outliers, leading to inflated “normal” intervals.

On the other hand, the moving window technique was found to be a better alternative in this case since it does not take into consideration the data set as a whole but only considers the variability in a local neighborhood for defining the threshold. Once again, a window size was chosen for each variable from a list of possible sizes {50, 500, 1000, 2000, 3000, and 5000}. The selection of an adequate window size was defined case by case and confirmed with plant engineers to reassure that the points removed were, in fact, abnormal. From Figure 7e, it is possible to validate the effectiveness of the moving window technique, which identified and removed most contextual outliers. The adaptive Hampel algorithm was therefore applied to all process variables.

### 5.3. Data Pre-Processing

Table 3 provides information about the number of samples, number of predictors, and levels of missing data for the different scenarios of resolution/synchronization considered in this study (see Section 3.3).

Since there were no non-operation periods to remove after the data cleaning stage, the number of samples remains the same as in the raw collected data. However, the amount of missing data has increased since the global and contextual outliers were replaced by blanks instead of removing the entire multivariate observation from the data set.

In all synchronized scenarios, the pre-processed data set has 243 new observations because, in these scenarios, the aggregation only takes place when there is a record of RON. Since there are 243 samples of RON, the data set after the synchronized resolution will have the same number of observations. Variable $X_{1}$ does not have missing records, but other variables may have, and this issue needs to be taken into consideration. 

As mentioned before, some predictive methodologies do not handle missing data. Therefore, a robust interpolative method was adopted to estimate missing records for each scenario. The imputation was carried out via a moving window median approach.

### 5.4. Prediction Accuracy Assessment and Comparison

As described previously, RMSE^test^ and R^2^_test_ were employed to evaluate the prediction capabilities of the different methods tested. The RMSE is the commonly adopted accuracy measure for estimating the standard error of prediction obtained for the different methods. The RMSE^test^ obtained are presented in Table 4. The corresponding results for the R^2^_test_ are presented in Appendix C.

Analyzing the results obtained and summarized in Table 4 and Table A2 (Appendix C), it is possible to verify that some methods present an adequate performance regarding prediction accuracy. Most of the regression methods present RMSE^test^ values near 0.5, being quite accurate for most practical purposes. The results also point to a certain advantage of using penalized regression methods, partial least squares, and kernel partial least squares with radial basis function, over the remaining linear and non-linear modelling approaches. This may be due to the existence of significant correlations between some process variables, such as temperatures in the reaction zone, which may lead to rather unstable models unless they are stabilized with some suitable technique, such as regularization or projection to latent spaces. This characteristic of the data overshadows the potential presence of non-linearity in the system and leads to the selection of methods that are able to cope with it rather than being capable of describing some mild curvature. This trend is reinforced by the stability of the process, which reduces the manifestation of non-linearity. Upon a closer inspection of the models, we could also verify that the variables found to be most important originated at the reaction and feed zones, which is consistent with existing Chemical Engineering background knowledge about phenomena taking place in this unit.

**Figure 8:**
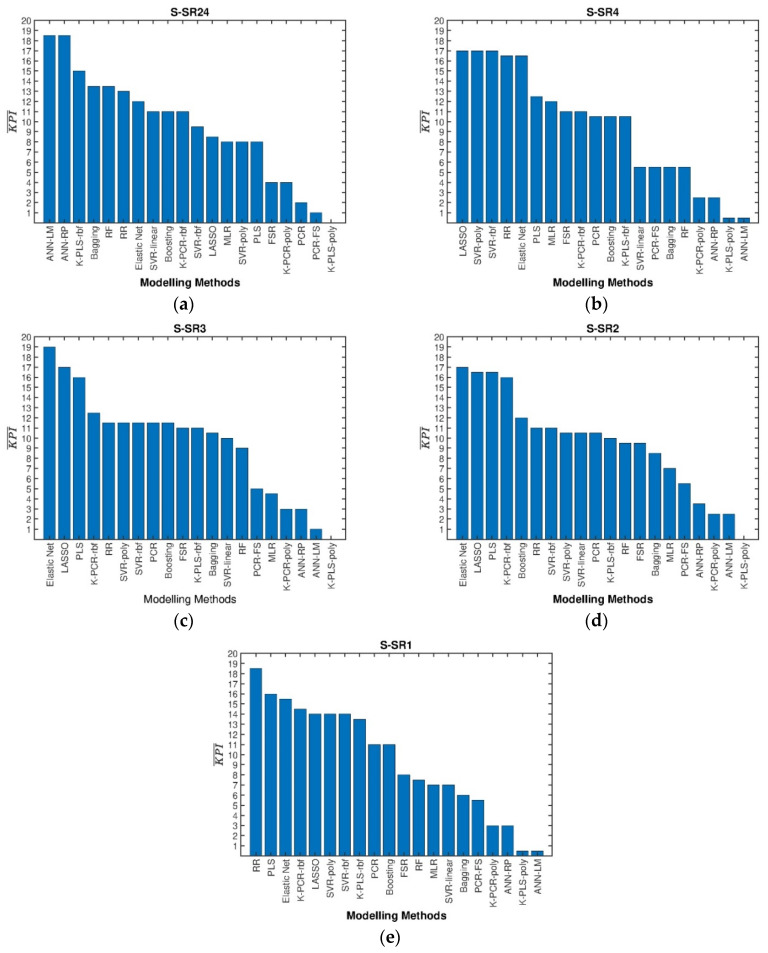
Results for all methods under analysis and for different granularity scenarios: (**a**) S-SR24; (**b**) S-SR4; (**c**) S-SR3; (**d**) S-SR2; (**e**) S-SR1.

The pairwise statistical hypothesis tests led to the ${\bar{\mathrm{KPI}}}_{m}$ scores presented in Figure 8 for the different resolution/synchronization scenarios.

These results confirm the superior performance achieved with the penalized regression methods over all the others in the prediction of RON in this unit, and in particular, the good performance of Elastic Net and LASSO.

**Figure 9:**
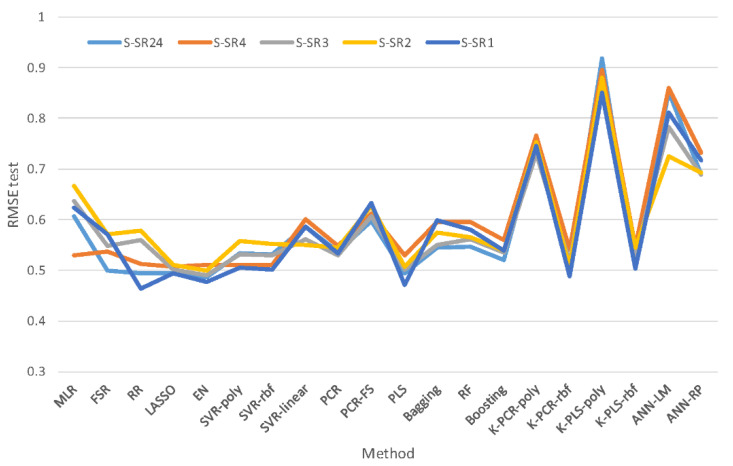
RMSE^test^for the 20 predictive methods in the five resolution/synchronization scenarios.

Regarding the scenarios of resolution/synchronization, it is possible to observe that there are no major trends, but some resolution levels tend to show better performance than others. In particular, some of the lowest RMSEs are obtained for the synchronized scenario with a granularity of 1 h, S-SR1 (namely for PLS, RR and EN; see Figure 9 for RMSE^test^ and Appendix C for R^2^_test_), and this may be due to the fact that the CCR plant has residence times that range between two and three hours. In general, several reasons may interfere with the definition of the best granularity to adopt, such as the level of unstructured variability present, the dynamic characteristics of the process, the delays between units, and the availability of data for certain variables, among others. For instance, a stable process with large unstructured variation and noise sources may favor the use of longer averaging windows (coarser granularity), whereas processes exhibiting clear, dynamic patterns in the process variables with lower levels of noise can be better described using a less granular representation of the data (finer granularity). The best compromise must be found case by case as if it was an additional tuning parameter of the models. Multi-granularity (or multiresolution) models could also be developed, where the granularity is defined for each variable under analysis [23,88].

## 6. Conclusions

In this work, a detailed data analytics workflow was presented and applied to address the challenging problem of predicting RON in the Catalytic Reforming Unit of an oil refinery, using only process data, and identifying the most relevant sources of RON variability. This workflow was implemented as a generic data analysis platform and includes data cleaning, data synchronization, and data resolution definition stages, together with extensive model testing and comparison.

A rich variety of predictive methods, representative of different classes of regression methodologies, was studied (twenty methods overall) and compared for the task of RON prediction. This comparison methodology was based on a Monte Carlo Double Cross-Validation approach to ensure an accurate and robust assessment of their relative predictive merits.

For the CCR data set, the best results were obtained for methods arising from the linear spectrum of predictive analytics, namely with ridge regression, elastic net and partial least squares. From the non-linear methods, kernel partial least squares with a radial basis function also presented interesting results, considering the several resolutions studied. The good process control of the CCR unit may be the reason why non-linearity is not so relevant here (as the process is not significantly perturbed) and, therefore, linear approaches that are able to cope with collinearity and can be applied with good accuracy and stability, were the selected solutions.

This result is important not only for the specific application under analysis but in a more general setting, as it underlines the importance of considering representatives from the full spectrum of predictive solutions when addressing complex industrial processes. In particular, it is not advised to assume that the most complex non-linear approaches, such as deep neural networks and others, despite the very good results achieved in many data-intensive applications, will necessarily translate such outstanding performance for industrial systems as well. Moreover, the same applies to any technique, as postulated (but often forgotten) in the celebrated NFL (“no free lunch theorem”) by David Wolpert published in 1996.

Regarding the different resolution/synchronization scenarios studied, the results point to the use of the synchronized scenario with a granularity of 1 h, S-SR1. Most of the data-driven methods tested with real plant data collected from the refinery led to predictions of RON values with reasonable accuracy. These results deserve particular consideration, given the existence of numerous unmeasured sources of variation in a large-scaled industrial process such as in a refinery, which introduces non-predictive components into the data, as well as possibly some missing elements and noise. From the refinery operation perspective, as transmitted by its plant engineers, the results obtained are promising, considering that only process variables are used to estimate RON, as well as the order of magnitude for what is considered from a practical industrial point of view as being an acceptable prediction error (equal or below 0.5). Therefore, the results achieved open good perspectives for future industrial applications, as RON is a critical process outcome, and the current methods to estimate its values are rather complex, expensive, and involve a long-time delay until the measurement becomes available.

In this work, it was shown that using a workflow composed of statistical and machine learning tools can indeed efficiently lead to quite good results in a relatively short time frame, even for rather complex problems, such as the prediction of RON values from process variables. These data-driven models can be instrumental in supporting process improvement efforts, namely regarding energy consumption, for instance, by avoiding excessive heating in the furnaces and heat exchangers at the inlet of the reactors, thus also reducing emissions levels and increasing the refinery’s bottom-line results.

## Figures and Tables

**Table 1:** Pseudo-code for the comparison framework adopted in this work.

1. For i = 1: $n_{\mathrm{MC}}$ (Number of Outer Cycles) perform:
Randomly split the complete data set into a training (80%) and testing set (20%) The training set is used to tune the hyperparameter(s) using 10-fold cross-validation (Inner Cycle). Estimate the model with the training set and the selected hyper-parameter(s) Predict the observation in test set and compute the Root Mean Squared Error (${\mathrm{RMSE}}_{i,m}^{\mathrm{test}}$, where m is the index of the method).
2. Apply a paired *t*-test to assess the statistical significance of the difference between the ${\mathrm{RMSE}}_{{1:N}_{\mathrm{MC}},m}^{\mathrm{test}}$ for all pairs of methods.
3. Using the *p*-values for paired statistical tests, compute the overall performance criteria:
Compute ${\bar{\mathrm{KPI}}}_{m}$ using Equation (4) Compute ${\bar{\mathrm{Rank}}}_{m}$ using Equation (5)

**Table 2:** Number of RON samples and the corresponding range, mean and standard deviation.

Property	Number of Samples	Property Values			
		Min.	Max.	Mean	SD
RON	243	96.80	102.50	100.38	0.97

**Table 3:** Number of samples and percentages of missing data in each scenario tested.

Resolution Scenario	Number of Samples	Number of Predictors	$\mathrm{Missing}\;\mathrm{Data}\;X_{1}\;(\%)$
Raw Data	1,048,320	41	0.02
After Cleaning	1,048,320	41	3.56
S-SR24	243	41	0.00
S-SR4	243	41	0.00
S-SR3	243	41	0.00
S-SR2	243	41	0.00
S-SR1	243	41	0.00

**Table 4:** Average RMSE^test^ (in test conditions) over all cross-validation trials for each regression method tested and in each scenario of resolution/synchronization.

Method	S-SR24	S-SR4	S-SR3	S-SR2	S-SR1
MLR	0.607	0.529	0.637	0.666	0.624
FSR	0.500	0.538	0.548	0.571	0.571
RR	0.494	0.513	0.560	0.578	0.464
LASSO	0.493	0.508	0.502	0.511	0.493
EN	0.486	0.510	0.490	0.500	0.477
SVR-poly	0.533	0.510	0.531	0.558	0.506
SVR-rbf	0.531	0.510	0.530	0.552	0.502
SVR-linear	0.586	0.600	0.561	0.551	0.585
PCR	0.537	0.548	0.530	0.544	0.533
PCR-FS	0.597	0.612	0.606	0.627	0.633
PLS	0.494	0.530	0.502	0.508	0.471
Bagging	0.545	0.595	0.550	0.574	0.599
RF	0.546	0.595	0.561	0.566	0.581
Boosting	0.520	0.559	0.535	0.539	0.539
K-PCR-poly	0.752	0.766	0.731	0.752	0.745
K-PCR-rbf	0.509	0.540	0.517	0.507	0.489
K-PLS-poly	0.918	0.896	0.856	0.880	0.851
K-PLS-rbf	0.510	0.547	0.532	0.544	0.504
ANN-LM	0.852	0.860	0.782	0.725	0.811
ANN-RP	0.690	0.732	0.690	0.692	0.718

**Figure A1:**
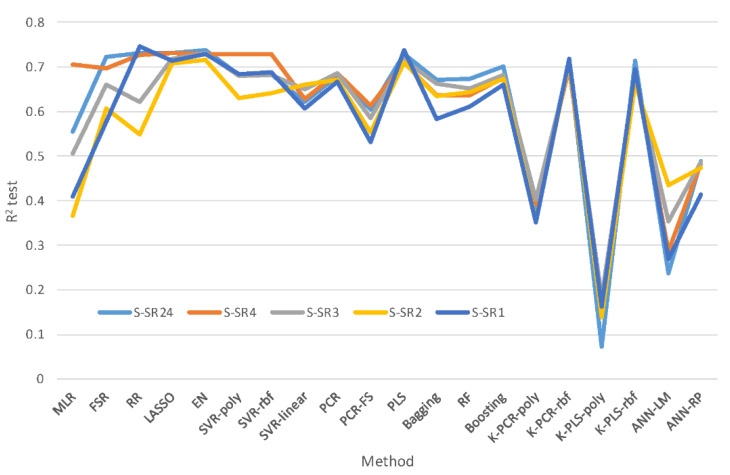
R^2^_test_ for the 20 predictive methods in the five resolution/synchronization scenarios.

## Data Availability

The data are not publicly available due to confidentiality restrictions related to sensible industrial information.

## Appendix A. A Brief Overview of Regression Methods Studied in This Work

In this work, we have explored 20 regression methods arising from different classes of predictive methods. Each class shares affinities in the estimation algorithms used and assumptions about the data structure or functional relationships. Next, we provide a short introduction to each method, together with references where more complete descriptions can be found. In addition to the more advanced methods, the classical Multiple Linear Regression (MLR) approach was included in this study for completeness since it is a well-known technique, widely used and implemented together with several variable selection methods considered in this work [16,46]. The MLR model, $Y=b_{0}+\sum_{j=1}^{p}b_{j}X_{j}+\varepsilon$, assumes that only the response variable carries a sizeable error, which is additive and homoscedastic (constant variance). The regression coefficients are found by least squares fitting, as described in Equation (A1):

(A1) $${\hat{b}}_{\mathrm{MLR}}=\underset{b={\left[b_{0}\ldots b_{P}\right]}^{\;T}}{\arg\min}\left\{\sum_{i=1}^{n}{\left(y_{i}-{\hat{y}}_{i}\right)}^{2}\right\}$$

where, ${\hat{b}}_{\mathrm{MLR}}$ is a vector containing the regression coefficients, n is the number of observations, $y_{i}$ is the $i^{th}$ observed response value and ${\hat{y}}_{i}$ is the respective estimated response. MLR faces problems when predictors present moderate-high levels of collinearity because the estimation of the regression coefficients becomes unstable (high variance). In that case, other approaches are available that lead to more stable predictions. Next, we present the other classes of methods considered and tested in this work.

***Variable Selection Methods.*** In variable selection methods, a subset of the predictor’s variables is selected according to a given criterion and is then used for estimating the model, while all the others are discarded. Forward stepwise regression (FSR) successively includes and excludes variables according to the p-value of a partial F-test [47,48,49], even though other information-theoretic measures can also be employed (e.g., AICc, BIC).

***Penalized Regression Methods.*** In the class of penalized regression methods, the regression coefficients are obtained by minimizing the sum of squared residuals while penalizing for the magnitude of the regression vectors (regularization). The penalization/regularization term is used to stabilize the estimation of the coefficients at the expense of introducing some bias in the estimation (the well-known bias-variance trade-off: a decrease in variance compensates for the higher bias of the predictions). This class of methods includes ridge regression (RR) [16,17], least absolute shrinkage and selection operator (LASSO) [15], and the elastic net (EN) [19,20]

***Latent Variable Methods*.** Variable selection methods assume that only some observed variables may influence the observed response (predictors sparsity) while others should be discarded. On the other hand, in the latent variable methods, it is assumed that the observed variability in both **X** and **Y** arises from a few underlying variables (latent variables), which are unobservable but can be inferred from linear combinations of the original variables. From this group of methods, three methodologies were analyzed: principal component regression (PCR) [50,51,52,53], principal component regression with a forward stepwise selection strategy (PCR-FS), and partial least squares (PLS) [54,55,56,57,58,59,60,61,62].

***Tree-Based Ensemble Methods*.** A regression tree approximates the relationship between the inputs and outputs by a piece-wise constant function, forming the building blocks for constructing the ensembles of predictors. In this class, three methods were used: Bagging of regression trees, Random Forests and Boosting of regression trees [19,63,64,65,66,67].

***Artificial Neural Networks*.** Artificial neural networks (ANNs), such as feed-forward neural networks, form a class of biologically inspired non-linear regression methodologies that mimic the learning processes taking place in the human brain [68,70,71,72,73]. ANNs are popular due to their ability to model complex non-linear functions. Usually, one hidden layer is enough to approximate continuous functions. However, as the complexity of the problems grows, it may be necessary to increase the number of hidden layers.

The most common training method for the ANN methodology is the backpropagation algorithm, and it will be adopted in this work. The choice for this algorithm is not arbitrary since it has been extensively studied and is a common choice in many practical applications, including in Chemical Engineering [74,75,76,77]. This algorithm consists of two phases: the forward propagation phase followed by the backward propagation phase [68,69]. We have considered several backpropagation algorithms to estimate the neural network parameters, including Levenberg–Marquardt backpropagation (LM) and resilient backpropagation (RP).

***Kernel PLS*.** Partial least squares (PLS) regression introduces the concept of latent variables to describe the linear multivariate relationship between the predictors’ matrix, **X**, and the response matrix, **Y**. However, it may be of interest to consider the presence of non-linear behaviour [78,79]. One way to bring non-linear modelling to the PLS scope is through kernelization. The basic idea is to map the data **X** into a high dimensional feature space Ƒ via a non-linear mapping φ and then perform a regression in this new space feature. This is the principle of Kernel PLS (KPLS) to estimate the relationship between **X** and **Y**. The selection of kernel function to adopt for the mapping is relevant, and in this work, we have considered two representatives: the Gaussian radial basis function, Equation (A2) and the polynomial, Equation (A3) [82].

(A2) $$K_{ij}=K\left(x_{i},x_{j}\right)=\exp\left(-\frac{{\left\|x_{i}-x_{j}\right\|}^{2}}{2\sigma^{2}}\right)$$

(A3) $$K_{ij}=K\left(x_{i},x_{j}\right)={\left(\langle x_{i},x_{j}\rangle +1\right)}^{p}$$

***Kernel PCR*.** Kernel PCR (KPCR) is similar to KPLS, but instead of using the NIPALS algorithm to select the latent variables, the method uses principal component analysis for obtaining the scores (principal components). The general idea for kernel PCR is again to map the original dataset into a higher-dimensional feature space, where it is possible to use PCA to create a linear relationship between the features, which are non-linearly related to the original input space [19,80,81,89]. For KPCR, we have used the same type of kernels as for KPLS: polynomial and Gaussian radial basis functions.

***Support Vector Machines Regression*.** Support Vector Machines Regression (SVR) is another machine learning method with the ability to handle non-linear relationships [83,84,85,86,87]. This methodology also projects data into a high dimensional feature space (by transforming the original variables with different kernel functions), penalizing the resulting complexity with a specific term added to the loss function, which also contains an ε–sensitive loss term.

## Appendix B. Details on Hyperparameters Selection for All Regression Methods

This appendix presents the range of the hyperparameter(s) used in the regression methods, as well as their selection strategy.

**Table A1:** Hyperparameter(s) range(s) for each method considered during the model training stage.

Method	Hyperparameter(s)	Possible Value(s)	Selection Strategy
MLR	-	-	-
FSR	$p_{\mathrm{in}}$ $p_{\mathrm{out}}$	0.05 0.10	-
PCR	$a_{\mathrm{PCR}}$	1:min(20,n,p)	10-fold cv
PCR-FS	$p_{\mathrm{in}}$ $p_{\mathrm{out}}$	0.05 0.10	10-fold cv
PLS	$a_{\mathrm{PLS}}$	1:min(20,n,p)	10-fold cv
RR	α γ	0 0.001; 0.01; 0.1; 1; 10	10-fold cv
LASSO	α γ	1 0.001; 0.01; 0.1; 1; 10	10-fold cv
EN	α γ	0; 0.167; 0.333; 0.500; 0.667; 0.833; 1 0.001; 0.01; 0.1; 1; 10	10-fold cv
BRT	$T_{\mathrm{BRT}}$	50; 100; 500; 1000; 5000	10-fold cv
RF	$T_{\mathrm{RF}}$	50; 100; 500; 1000; 5000	10-fold cv
BT	$T_{\mathrm{BT}}$	50; 100; 500; 1000; 5000	10-fold cv
SVR-linear	$\varepsilon_{\mathrm{linear}}$	0.001; 0.005; 0.01; 0.05; 0.1	10-fold cv
SVR-poly	$\varepsilon_{\mathrm{rbf}}$	0.001; 0.005; 0.01; 0.05; 0.1	10-fold cv
SVR-rbf	$\varepsilon_{\mathrm{poly}}$	0.001; 0.005; 0.01; 0.05; 0.1	10-fold cv
K-PCR-poly	$a_{\mathrm{PCR}}$ $p_{\mathrm{poly}}$	1:30 2; 4; 6; 8; 10	10-fold cv
K-PCR-rbf	$a_{\mathrm{PCR}}$ $p_{\mathrm{rbf}}$	1:30 0.1; 1; 10; 50; 100; 300; 1000	10-fold cv
K-PLS-poly	$a_{\mathrm{PLS}}$ $p_{\mathrm{poly}}$	1:30 2; 4; 6; 8; 10	10-fold cv
K-PLS-rbf	$a_{\mathrm{PCR}}$ $p_{\mathrm{rbf}}$	1:30 0.1; 1; 10; 50; 100; 300; 1000	10-fold cv
ANN-LM	layer $n_{\mathrm{LM}}$	1 5; 10; 15	10-fold cv
ANN-RP	layer $n_{\mathrm{RP}}$	1 5; 10; 15	10-fold cv

## Appendix C. Additional Results for the Comparison Study

The coefficient of determination $R^{2}$ between predicted and observed responses, obtained over a large number of cross-validation trails, assesses the methods’ prediction accuracy in terms of a normalized parameter. The results obtained for the different methods studied are summarized in Table A2 and Figure A1.

**Table A2:** Average R^2^_test_ (in test conditions) over all cross-validation trials for each regression method tested and in each scenario of resolution/synchronization.

Method	S-SR24	S-SR4	S-SR3	S-SR2	S-SR1
MLR	0.555	0.705	0.505	0.366	0.410
FSR	0.722	0.697	0.660	0.607	0.577
RR	0.730	0.727	0.622	0.549	0.745
LASSO	0.730	0.732	0.719	0.708	0.713
EN	0.738	0.729	0.732	0.717	0.728
SVR-poly	0.681	0.728	0.680	0.630	0.683
SVR-rbf	0.683	0.729	0.681	0.641	0.689
SVR-linear	0.622	0.627	0.649	0.661	0.606
PCR	0.680	0.687	0.686	0.670	0.666
PCR-FS	0.605	0.612	0.586	0.553	0.531
PLS	0.729	0.709	0.717	0.709	0.738
Bagging	0.672	0.636	0.663	0.635	0.583
RF	0.674	0.637	0.652	0.644	0.610
Boosting	0.701	0.676	0.681	0.674	0.661
K-PCR-poly	0.376	0.392	0.403	0.353	0.351
K-PCR-rbf	0.714	0.696	0.702	0.711	0.718
K-PLS-poly	0.073	0.166	0.185	0.138	0.163
K-PLS-rbf	0.713	0.687	0.681	0.662	0.695
ANN-LM	0.238	0.287	0.353	0.436	0.269
ANN-RP	0.488	0.485	0.486	0.474	0.413
